# Adherence, satisfaction, and quality of life in Wilson disease patients after switching to trientine tetrahydrochloride: observational data from a dual cohort study

**DOI:** 10.3389/fphar.2025.1515686

**Published:** 2025-10-23

**Authors:** Valentina Medici, Nora Cazzagon, Pier M. Battezzati, Andrea Crosignani, Alberto Civolani, Emanuela Bonaiuto, Renata D’Incà, Emanuela Vargiu, Fabio Tedone, Stefania Lopatriello, Massimo Zuin

**Affiliations:** ^1^ Division of Gastroenterology and Hepatology, Department of Internal Medicine, School of Medicine, University of California, Davis, CA, United States; ^2^ Department of Surgery, Oncology and Gastroenterology, Rare-Liver ERN, Azienda Ospedale – Università Padova, University of Padova, Padova, Italy; ^3^ Department of Health Sciences, ASST Santi Paolo e Carlo, Rare-Liver ERN, Polo Universitario San Paolo, University of Milan, Milano, Italy; ^4^ UOC Gastroenterology AOU Cagliari Policlinico “Duilio Casula”, Cagliari, Italy; ^5^ Helaglobe, Firenze, Italy; ^6^ Department of Health Sciences, University of Milan, Milano, Italy

**Keywords:** Wilson disease, trientine tetrahydrochloride, copper, patient-reported outcomes, treatment adherence, Quality of life (QoL)

## Abstract

**Background:**

Wilson disease (WD) is a rare genetic disorder requiring lifelong treatment. Adherence to therapy is thus of utmost importance. We aimed to assess the change in treatment adherence and satisfaction in adult patients with WD after transitioning to trientine tetrahydrochloride (TETA 4HCl), a first-of-its-kind investigation.

**Methods:**

Observational, Italian multicenter study of adult patients switched to TETA 4HCl by their treating physicians. Eligibility criteria included WD patients either already switched (up to a maximum of 9 months before enrolment; retrospective-prospective [RP] cohort) or about to be switched (within 3 months following enrolment; prospective [P] cohort). Adherence was assessed by pill counts, self-reported Morisky Medication Assessment Scale and 24-h urinary copper excretion (UCE) values within expected range. Patient reported Experiences and Satisfaction with Medications and health related quality of life questionnaires were completed at baseline (T0) and after 3 months (T1).

**Results:**

25 patients were enrolled: 9 in the RP and 16 in the P cohort, with median (IQR) age and time since diagnosis was 43.0 [(30.0, 52.0)] years and 24.0 [(14.0, 30.0)] years respectively. At T0, 40% of patients self- reported adherence according to MMAS and 58.3% were considered adherent according to UCE values. At T1, values increased to 64% (for both MMAS and UCE). Pill count (T1) suggested that 64% could be considered adherent. On a ten-point Likert scale from −5 to +5, median (IQR) satisfaction score was 4.0 [(4.0, 5.0)]. All quality-of-life (QoL) domains improved between T0 and T1.

**Conclusion:**

Adults with WD on maintenance therapy following a switch in therapy to TETA 4HCl some improvements in adherence, satisfaction and QoL were observed.

## Introduction

Wilson disease (WD) is a rare autosomal recessive genetic disorder with an estimated prevalence of 3.3 per 100,000 individuals (Ala et al., 2007). The multi-organ disease is caused by mutations affecting the *ATP7B* gene, which encodes a protein expressed mainly in the liver that regulates copper transport within and excretion from hepatocytes (Tanzi et al., 1993). Genetic mutations can reduce biliary copper excretion, resulting in liver copper accumulation. Once storage capacity is exceeded, copper is released into the bloodstream and may accumulate in multiple organs, including brain, causing varied clinical presentations (Schi et al., 2023). Hepatic signs and symptoms, including elevation of liver enzymes or signs of advanced liver disease and portal hypertension (ascites, esophageal varices bleeding and hepatic encephalopathy) typically characterize WD, but neuro-psychiatric symptoms, or a combination of these may be encountered.

Diagnosing WD is challenging due to the various combinations of signs and symptoms and the need for reliable diagnostic tests, leading to frequent delays in diagnosis (Bruha et al., 2011). If left untreated, WD can be fatal, but prompt initiation of pharmacological therapy can halt disease progression and increase life expectancy (Bruha et al., 2011).

Current therapeutic options include copper-chelating drugs, such as D-penicillamine (DPA) and trientine, as well as zinc salts, to induce and maintain a negative copper balance in the body. Chelating agents remain the principal therapy with zinc salts reserved for maintenance therapy and asymptomatic or neurologically affected patients (Weiss et al., 2013). Neurologic worsening may occur following the initiation of all therapies (most notably, penicillamine) and close monitoring is recommended (Roberts and Schilsky, 2023).

Trientine has a more favorable safety profile than DPA, which has poorer tolerability and more frequent adverse events (Merle et al., 2007; European Association for Study of Liver, 2025). A recent prospective head-to-head randomized trial demonstrated the non-inferiority of trientine tetrahydrochloride to DPA (Schilsky et al., 2022). As reported in the CHELATE Study: “the primary endpoint, assessed 24 weeks after randomization, was NCC by speciation assay. Using this NCC speciation method, TETA4 was determined to be non-inferior to penicillamine in patients who were penicillamine tolerant at 24 weeks of treatment” (Schilsky et al., 2022).

Trientine tetrahydrochloride (TETA 4HCl; Orphalan SA) (European Medicine Agency, 2017) is a scored tablet, stable at ambient temperature with pharmacokinetic data demonstrating that it is more rapidly absorbed and provides greater systemic exposure of the active moiety, trientine (TETA), compared with trientine dihydrochloride (TETA 2HCl) (Weigand and Corsini, 2022).

WD requires lifelong therapy, at least twice daily, separated from meals, making adherence to therapy challenging. The consequences of non-adherence are potentially harmful, including hepatic, neurologic worsening, liver failure and death (Jacquelet et al., 2018; Masełbas et al., 2019).

The common indications for switching therapies in WD are side effects more frequently, autoimmune disorders, skin rashes, or bone marrow suppression associated with DPA, lack of efficacy, poor adherence or unavailability of medications. Low adherence to WD treatment occurs in one-fourth to one-third of WD patients (Masełbas et al., 2019; Jacquelet et al., 2021). Asymptomatic patients, for whom the perceived need for taking medications regularly may not be evident, are at high risk of non-adherence that may lead to discontinuation and worsening of disease (Jacquelet et al., 2021). There is currently no published data describing the impact of switching therapy on patient adherence in WD. This study aimed to investigate the adherence and satisfaction of patients with WD at two points during a switch in therapy and after switching therapy to TETA 4HCl.

## Methods

This observational, multicentre study with retrospective and prospective components was carried out in three Italian hospitals (Milan [coordinating centre], Padua, and Cagliari) between January 2021 and March 2023. The study protocol was approved by the ethics committee at each centre. The inclusion criteria were: patients with a confirmed diagnosis of WD (Leipzig score ≥4), over 18 years of age, and switch to TETA 4HCl as per clinical indications (including side effects or lack of improvement of copper metabolism parameters, such as 24 h urinary copper) by treating physician. Patients not switched to TETA 4HCl, were not eligible. All patients provided written informed consent.

Two cohorts formed the study population:

• The Retrospective-prospective (RP) cohort, included all patients who had started TETA 4HCl therapy no more than 9 months before the screening visit; the screening visit was registered as baseline (T0).
• The Prospective (P) cohort, included all patients identified by physicians suitable for and intending to switch therapy to TETA 4HCl after and within 3 months of the screening visit. In this cohort, the baseline (T0) time was the moment of new therapy initiation.

For the RP cohort, data were collected at three time points: at the switch time (ST), at baseline (T0), and at the first visit 3 months after the baseline visit (T1). For the P cohort, data were collected at two time points: at baseline/switch time (T0 = ST) and first visit 3 months after switch (T1).

The starting dose of TETA 4HCl would usually correspond to the lowest dose in the recommended dose range (between 450 mg/day and 975 mg/day; 3–6.5 film-coated tablets divided in 2-4 doses) and the dose should subsequently be adapted according to the patient’s clinical response. In order to maximize the assessment of adherence to prescribed therapy we adopted three methods: pill count at T1, administration of the Morisky Medication Adherence Scale (MMAS) questionnaire and measurement of 24-h urinary copper excretion (UCE) values at T0 and T1 (Colivicchi et al., 2010; Treibich et al., 2017; Morisky et al., 1986).

Pill count was measured as the percentage of pills returned at T1 compared to those dispensed at T0. Individuals were classified as “adherent” if they took more than 80% of the prescribed medication, “partially adherent” if they took between 20% and 80%, and “nonadherent” if they took less than 20% (Colivicchi et al., 2010).

In the RP cohort, the shorter version of the MMAS-4 questionnaire (Menditto et al., 2015) was administered due to a higher likelihood of errors in reporting recollection of past events and patients were classified “adherent” with a MMAS- scoring 3 and 4, “non-adherent” with lower scores. In the P cohort, the complete MMAS-8 questionnaire was administered and patients were categorized into three groups: “highly adherent” with a score equal to 8, “moderately adherent” with a score from 6 to 7, and “poorly adherent” with a score lower than 6.

The use of MMAS-4 in the RP cohort reflects the retrospective nature of the data collection, where memory recall bias was a concern. This difference in scale application is acknowledged as a limitation of the study.

UCE levels reflect a combination of adherence, dosing and chelating efficiency; values above or below the target range may provide additional information alongside pill counts and patient reported measures of adherence. Urinary copper excretion was collected as part of routine clinic visits, with values recorded at T0 and T1. Patients were considered adherent if their UCE values were within the reference range for the ongoing treatment at the time of the visit (Schilsky, 2017). The reference ranges of UCE used were: 150–500 µg/24 h (for chelation therapy) and for zinc therapy, <100 µg/24 h (Schi et al., 2023). No reference range of UCE for patients under combination was identified.

The secondary objectives included gathering patient-reported safety data and assessing patient satisfaction through the administration of the Patient Experiences and Satisfaction with Medications (PESaM) questionnaire (Kimman et al., 2019), as well as evaluating health related quality-of-life using the SF-36 questionnaire (Burholt and Nash, 2011). In detail, the PESaM questionnaire investigated, on scales from 1 to 5 or from −5 to 5, how patients perceive the efficacy, presence of side effects, usability, and overall satisfaction with the therapy compared to the pre-switch therapy. The results of the PESaM were calculated so that higher values corresponded to greater satisfaction with the new therapy.

The SF-36 scores were transformed to a linear scale from 0 to 100, where higher values indicate better wellbeing. All consecutive patients meeting eligibility criteria were enrolled and followed for at least 3 months. Demographic characteristics and treatment patterns data were collected as recorded in the clinical report forms by the investigators.

Patient characteristics were described by means of frequencies and percentages for categorical variables or by means (SD), median (IQR) for parametric and non-parametric continuous data. Mann-Whitney test for continuous variables and Fisher’s exact test for categorical variables were used to compare patient characteristics between cohorts. Mann-Whitney’s test for continuous variables and McNemar’s test for categorical variables were used to compare differences between T0 and T1 timepoints. Data was analyzed with Spearman’s correlation test according to factors related to patient, clinical condition, treatment, and socioeconomic status. The statistical significance threshold was set at p < 0.05. All statistical analyses were performed using Stata 16 (Stata Corporation, College Station, TX, United States).

## Results

A total of 25 patients (14 females) were enrolled, 9 in the RP cohort (36%) and 16 in the P cohort (64%). Demographic data are presented in Table 1. Table 2 contains details of individual results and UCE values at each visit.

**TABLE 1:** Patient characteristics.

	Retrospective- prospective (RP) N = 9	Prospective (P) N = 16	Overall N = 25
Female (n)	6	8	14
Age, years	37.0 [28.0,49.0]	45.5 [34.5,53.2]	43.0 [30.0,52.0]
Weight, Kg	64.0 [56.0,67.0]	63.0 [53.0,73.0]	64.0 [54.0,70.0]
Family status (n)			
With partner	5	7	12
Alone	1	0	1
With relatives	3	8	11
Other	0	1	1
Patients with comorbidities (n)	2 (*)	6 (**)	8
Educational status (n)			
High school diploma or less	4	5	9
Secondary high school diploma	1	4	5
University degree	4	7	11
Professional status (n)			
Employee	7	11	18
Not working	0	2	2
Retired	2	3	5
Years since diagnosis	18.0 ([14.0,24.0])	26.0 [15.5,30.5]	24.0 [14.0,30.0]
Years since diagnosis (%)			
0–14 years	28.0%	25.0%	28.0%
15–29 years	44.0%	37.5%	44.0%
30–45 years	28.0%	37.5%	28.0%
Pre-switch therapy (n)			
Zinc	5	8	13
Chelation	3	7	10
Combination	1	1	2
Exposition time to TETA 4HCl before T0 (n)			
0 month	0	16	16
1–5 months	3	0	3
6–9 months	6	0	6

Continuous variables are summarized as median (interquartile range).

(*) Psoriatic arthritis, cholelithiasis and nephrolithiasis.

(**) Atrial fibrillation, dyslipidemia, thoracic myelopathy, asthma, edematous gastritis, alcohol abuse and HIV.

Abbreviations: RP, retrospective-prospective; P, prospective; TETA, 4HCl, Trientine Tetrahydrochloride.

**TABLE 2:** Individual results and UCE values at each visit.

ID	Cohort	Pre-switch therapy	ST^a^ UCE (mcg/24 h)	T0 UCE (mcg/24 h)	T1 UCE (mcg/24 h)	Adherence by MMAS at T0	Adherence by MMAS at T1	Adherence by pill count	Adverse events at T0	Adverse events at T1
1	P	Penicillamine	201	201	610	Moderately adherent	Very adherent	Partially adherent	Headache	
3	P	TETA 2HCl/Zinc	49	49	166	Moderately adherent	Moderately adherent	Adherent	Gastritis	
4	P	Zinc	50	50	76	Moderately adherent	Very adherent	Partially adherent		
5	P	TETA 2HCl	96	96	95	Poorly adherent	Moderately adherent	Partially adherent		
7	P	Zinc	354	354	535	Poorly adherent	Very adherent	Partiall y adherent		
10	P	Zinc	76	76	178	Very adherent	Very adherent	Adherent	Gastritis	
13	P	Zinc	275	275	350	Moderately adherent	Very adherent	Adherent		
14	P	Zinc	19	19	211	Poorly adherent	Moderately adherent	Adherent	Gastritis	
15	P	Zinc	63	63	196	Poorly adherent	Moderately adherent	Adherent	Gastritis	Gastritis
16	P	Zinc	36	36	156	Poorly adherent	Poorly adherent	Adherent		
17	P	Penicillamine	580	580	233	Poorly adherent	Moderately adherent	Adherent	Cutis laxa	
18	P	Penicillamine	19	19	211	Very adherent	Very adherent	Adherent		
22	P	Penicillamine	317	317	490	Poorly adherent	Moderately adherent	Adherent	Cutis laxa	
23	P	Penicillamine	76	76	140	Very adherent	Very adherent	Adherent	Gastritis	
24	P	Penicillamine	757	757	21	Very adherent	Very adherent	No data	Gastritis	
25	P	Zinc	198	198	138	Poorly adherent	Poorly adherent	No data	Headache	Headache
2	RP	TETA 2HCl	106	161	182	Adherent	Adherent	Adherent		
6	RP	Zinc	71	65	593	Adherent	Adherent	Adherent	Gastritis	
8	RP	Zinc	175	175	346	Non adherent	Adherent	Adherent	Gastritis	
9	RP	Zinc	102	275	843	Adherent	Adherent	Adherent		
11	RP	TETA 2HCl	96	354	335	Non adherent	Adherent	Partially adherent		
12	RP	Zinc	23	35	97	Non adherent	Adherent	Partially adherent		
19	RP	TETA 2HCl	130	175	271	Adherent	Non adherent	Adherent		
20	RP	Zinc	102	195	313	Adherent	Adherent	Poorly adherent		
21	RP	TETA 2HCl/Zinc	414	272	334	Adherent	Adherent	Adherent		

Pill count was measured as the percentage of pills returned at T1 compared to those dispensed at T0. “Adherent” patients took more than 80% of the prescribed medication, “partially adherent” took between 20% and 80%, and “nonadherent” took less than 20%.

In the RP, cohort, MMAS-4, was administered. Patients were classified “adherent” with a score 3 and 4, “non-adherent” with lower scores. In the P cohort, MMAS-8, was administered and patients were categorised into three groups: “highly adherent” with a score equal to 8, “moderately adherent” with a score from 6 to 7, and “poorly adherent” with a score lower than 6.

^a^ ST = T0 for P cohort.

Abbreviations: RP, retrospective-prospective; P, prospective; TETA, 2HCl, Trientine Dihydrochloride; TETA, 4HCl, Trientine Tetrahydrochloride; ST, switch time; UCE, urinary copper excretion; MMAS, morisky medication adherence scale.

The median age (IQR) and time since diagnosis were 43.0 [(30.0, 52.0)] and 24.0 [(14.0, 30.0)] years, respectively. Eighteen patients (72%) were employed, and 24 participants (96%) reported living with their partner or family members; only 1 patient (RP cohort) reported living alone.

Before switching to TETA 4HCl therapy, 13 (52%) patients were treated with zinc salts, 6 patients were treated with penicillamine, 4 with TETA 2HCl, and 2 with a combination of TETA 2HCl and zinc. In the P cohort, for the 8 patients treated with zinc salts the median dosage was 150 mg, for the 6 patients treated with penicillamine the median dosage was 600 mg, one patient was treated with TETA 2HCl 900 mg, and 1 patient was treated with a combination of TETA 2HCl 500 mg and zinc 750 mg. In the RP cohort, the median number of months (IQR) of treatment with TETA 4HCl before enrolling was 6.8 [(5.0, 8.5)] months. No significant differences were found in the patient demographics reported in Table 1 between the RP and P cohorts.

### Pill count

The mean (SD) posology (rounded up to the whole tablet) of TETA 4HCl was 4 (Ala et al., 2007) tablets per day. Two patients did not provide the returned tablet number at the clinic visit and their data were considered missing. In the overall cohort, based on the pills returned at T1, 16/23 patients (69.6%) were adherent (intake of more than 80%), with 10/16 patients in the P cohort and 6/9 in the RP cohort. Six patients (24%) were partially adherent, with 4/16 in the P cohort and 2/9 in the RP cohort. Additionally, 1 patient (4%) in the RP cohort was non-adherent.

In the subgroup of patients classified as partially adherent, 2 patients in the RP cohort showed intake less than 50% (20% and 46.3%, respectively), while 4 patients in the P cohort exhibited intake of tablets ranging from 59% to 75%.

We observed a significant correlation between years since WD diagnosis and pill count (r = 0.47, p = 0.0257). Specifically, according to the pill count, patients diagnosed for over 20 years were more likely to be adherent (intake greater than 80% of prescribed tablets) than those diagnosed with WD for less than 20 years (10 patients with WD for over 20 years vs. 6 patients with WD for less than 20 years, p = 0.033).

### MMAS questionnaires

**FIGURE 1:**
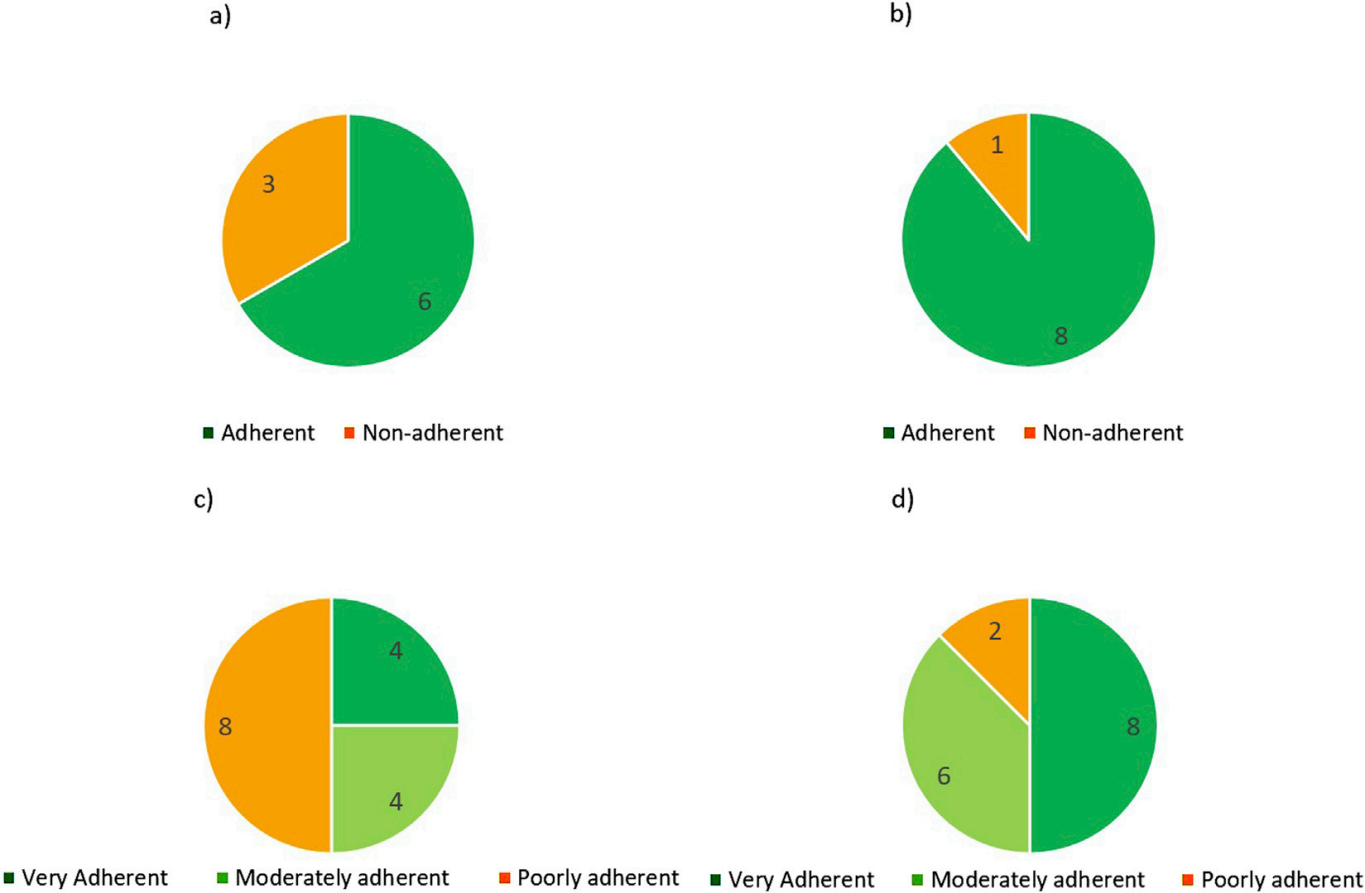
MMAS-4 scores at T0 **(a)** and T1 **(b)** and MMAS-8 scores at T0 **(c)** and T1 **(d)**.

According to the results of the MMAS-4 questionnaire in the RP cohort, 3 patients (33.3%) were considered non-adherent at T0, on TETA 4HClbut all became adherent at T1 (Figure 1), although the results are not statistically significant. In the P cohort, at T0, 8 patients (50%) were classified as poorly adherent and this reduced by 75% to 2 patients at 3 months follow up. The number of patients classified as very adherent increased by 100% from 4 at T0 to 8 at 3 months (T1). Two patients remained poorly adherent. No patient reported a worse adherence score at T1 than at T0.

### 24-h urinary copper excretion (UCE) values

**FIGURE 2:**
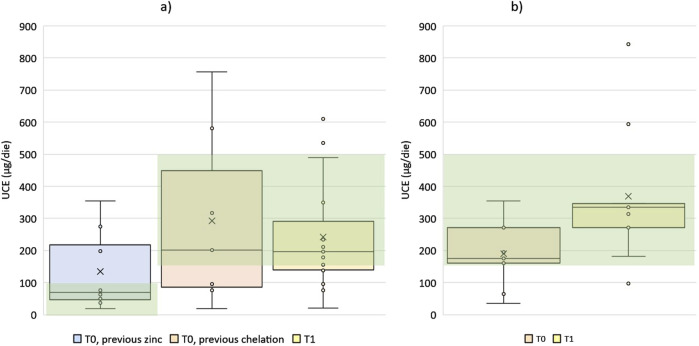
Distribution of UCE values for P cohort **(a)** and RP cohort **(b)** at timepoints and therapeutic target ranges (green background). Note, the patient in P cohort under combination at T0 is not reported in this Figure.

The maintenance range for zinc therapy was used as a reference at ST for patients previously treated with zinc in both P and RP cohorts (Table 3). For the only 2 patients (1 in P cohort and 1 in RP cohort) previously under combination, adherence was not assessed at time ST as no reference maintenance range was identified. For patients in P cohort, T0 coincides with ST. For patients in RP cohort at T0 and T1 and for patients in P cohort at T1, the maintenance range for chelation therapy was used as a reference. Median UCE values and number of patients considered adherent are reported in Table 3 and showed in Figure 2.

**TABLE 3:** UCE values at ST, T0 and T1 grouped by pre-switch therapy.

Cohort	Pre- switch therapy (n)	UCE at ST median µg/24 h (IQR)	n (%) in adherenc e range ST^a^	UCE at T0 median µg/24 h (IQR)	n (%) in adherence range T0^a^	UCE at T1 median µg/24 h (IQR)	n (%) in adherence range T1	p value UCE T0 vs. T1	Median variation T0 vs. T1 (%)
P	Chelation (7)	201.0 [(86.0, 448.5)]	2 (25%)	201.0 [(86.0, 448.5)]	2 (25%)	211.1 [(117.5, 361.5)]	4 (57.1%)	0.7422	+5.0%
	Zinc (8)	69.5 [(46.5, 217.2)]	5 (62.5%)	69.5 [(46.5, 217.2)]	5 (62.5%)	187.0 [(151.5, 245.7)]	5 (62.5%)	0.0234	+169.1%
	Combination (1)	49.0	-^b^	49.0	-^b^	166.0	1 (100%)	-	+238.7%
RP	Chelation (3)	106.0 [(101.0, 118.0)]	0 (0.0%)	175.0 [(168.0, 264.5)]	3 (100%)	271 [(226.5, 303.0)]	3 (100%)	0.2500	+54.9%
	Zinc (5)	102.0 [(71.0, 102.0)]	2 (40%)	175.0 [(65.0, 195.0)]	3 (60%)	346.0 [(313.0, 593.0)]	2 (40%)	0.0625	+97.7%
	Combination (1)	414	-^b^	272.0	1 (100%)	334.0	1 (100%)	-	+22.8%

Switch time (ST) values for P cohort are the same at T0 (T0 = ST, in P cohort).

^a^ For P Cohort at T0 = ST and for RP cohort at ST, UCE adherence range depends on previous therapy (zinc or chelation). For RP Cohort at T0, only chelation UCE adherence range was considered.

^b^ There was not found UCE, adherence range for patients in combination.

Abbreviations: IQR, interquartile range expressed as [1st quartile, 3rd quartile]; RP, retrospective-prospective; P, prospective; ST, switch time; UCE, urinary copper excretion.

At baseline (T0), 14 patients (56%) were classified as adherent. Specifically, 7 of them were in RP cohort, 2 in P cohort and previously treated with chelation, 5 in P cohort and previously treated with zinc.

At T1, 15 patients (60%) were classified as adherent, 6 in the RP cohort and 9 in the P cohort. In addition, the patient in P cohort receiving combination before the switch was classified as adherent at time T1.

The slight increase in the proportion of patients classified as adherent by UCE observed from T0 to T1 (56%–60%), was not statistically significant.

It is worthy to note that, in RP cohort, at switch time only 2/8 patients (25%) were classified as adherent according to the recommended UCE values ranges (Table 3). After switching to TETA 4HCl, the number of adherent patients increased to 7/9 patients (77.7%) at T0 and 6/9 (66.6%) at T1. Finally, we compared the adherence distributions estimated by pill count, MMAS questionnaire, and UCE values at T1.

For pill count, we considered two categories: “adherent” and “non-adherent” (i.e., those classified as “partially adherent” or “non-adherent”). For MMAS-8, we considered two categories: “adherent” and “non-adherent” (i.e., those classified as “moderately adherent” or “poorly adherent”). Regarding UCE values, we categorized patients into “adherent” and “non-adherent” (i.e., those with UCE values below or over the maintenance range for chelation therapy).

A correlation between pill adherence and UCE levels was noted for both the overall cohort and the P cohort (r = 0.51 p = 0.026 and r = 0.84 p = 0.005, respectively) but not for the RP cohort (r = 0.04; p = 0.762). No significant relationship between pill adherence and MMAS or UCE values and MMAS was found.

### Patient satisfaction

The PESaM questionnaire, used to assess patient’s satisfaction with the therapy was available in all but one patient (Tables 4–6). PESaM results showed a significant improvement in the usability domain (p = 0.0214), indicating a greater perceived simplicity for the patients in managing their therapy (Table 4).

**TABLE 4:** PESaM results.

Domain (min and max scores)	T0			T1			p value T0 vs. T1
	Median	IQR	Number of answers	Median	IQR	Number of answers	
Perceived Effectiveness (1,5)	4.0	[3.4,5.0]	25	3.9	[3.4,4.8]	24	0.9249
Perceived Satisfaction Effectiveness (−5,5)	4.0	[0.8,4.0]	25	4.0	[3.0,5.0]	24	0.2417
Adverse Event Weight (1,5)	2.7	[2,4.1]	12	3.2	[3.1,3.3]	2	NA
Impact Of Adverse Event (−5,5)	−1.5	[-3.2,0.2]	12	2.0	[1.0,3.0]	2	NA
Ease of use (1,5)	4.3	[3.7,4.8]	25	5.0	[4.3,5.0]	24	0.0214
Overall Satisfaction (-5,5)	4.0	[-1.3,5.0]	25	4.0	[3.5,5.0]	24	0.1847

Moreover, a non-significant improvement of the domain of satisfaction for the therapy safety, assessed as the impact of adverse events, was also observed (Table 4). Only the domain related to perceived efficacy showed a substantially similar median value at T1 and T0 (4 vs. 3.88; p = 0.9249).

Compared to the pre-switch therapy, more than 60% of respondents rated the perception of adverse events and ease of use with the highest satisfaction (score = 5) (Table 5). For the other two domains (perceived efficacy and overall satisfaction with the new treatment compared to the previous one), maximum satisfaction was reported by over 1 in 3 respondents, and in general, the median value was very high (Bruha et al., 2011) with very low dispersion (Table 6).

**TABLE 5:** Percentages of highest values on the individual scale PESaM domains.

Domain (min and max scores)	Minimum score	Number of answers vs. total answers	Percentage	Maximum score	Number of answers vs. total answers	Percentage
Perceived effectiveness in comparison with previous treatment (1,5)	1	1/24	4.2%	5	8/24	33.3%
Perceived Side effects in comparison with previous treatment (1,5)	0	1/23	4.3%	5	16/23	69.6%
Ease of use in comparison with previous treatment (1,5)	3	3/23	13.0%	5	14/23	60.9%
Overall satisfaction in comparison with previous treatment (−5,5)	−5	1/23	4.3%	5	9/23	39.1%

The results were calculated so that higher values corresponded to greater satisfaction with the new therapy.

**TABLE 6:** Evaluation towards pre-switch treatment.

Domain (min and max scores)	T1		
	Median	IQR	Number of answers
Efficacy compared with previous treatment (1,5)	4.0	[3.0,5.0]	24
Side effects compared with previous treatment (1,5)	5.0	[4.0,5.0]	23
Ease of use compared with previous treatment (1,5)	5.0	[4.0,5.0]	23
Satisfaction compared with previous treatment (−5,5)	4.0	[4.0,5.0]	23

Abbreviations: IQR, interquartile range expressed as [1st quartile, 3rd quartile].

### Safety

Twelve patients self-reported they experienced adverse events at T0 (2 in the RP cohort and 10 in the P cohort). At time T1, only 2 patients (P cohort) reported experiencing adverse events. All of them also experienced adverse events at time T0. For each patient self-reporting adverse event at T0 or T1, the type and severity of adverse events was explored by analysing physician’s notes. In the RP cohort, 2 out 9 patients experienced gastritis at T0 but experienced no treatment-emergent adverse events at T1. In the P cohort, 10 out of 16 patients self-reported adverse events at T0. Two patients with cutis laxa had been previously treated with penicillamine. Two patients with headaches have been previously treated with penicillamine and zinc. Six patients with gastritis had been previously treated with zinc (3 patients), penicillamine (2 patients) and a combination of zinc and TETA 2HCl (1 patient).

Two patients, previously treated with zinc, exhibited improvement in the severity of adverse events reported at T0 (gastric symptoms and headache, respectively).

Considering the RP and P cohorts as a whole, treatment with TETA 4HCl was well tolerated: no patient reported drug-related side effects and a regression or improvement of the adverse events induced by zinc or penicillamine was observed, taking also into account that reduced side effects may result from withdrawal of the prior treatment.

### Quality of life

Despite the excellent quality of life at baseline, significant improvements in the physical function (p = 0.0097) and role emotional (p = 0.0256) domains were observed. Non-significant improvements were observed in the other domains (Table 7).

**TABLE 7:** SF36 results.

Overall sample									p
Domain	T0				T1				
	Median	IQR	Min	Max	Median	IQR	Min	Max	
Physical Function	100.0	[90.0,100.0]	15.0	100.0	100.0	[94.0,100.0]	30.0	100.0	0.0097
Role Function	100.0	[100.0,100.0]	0.0	100.0	100.0	[75.0,100.0]	0.0	100.0	0.4262
Role Emotional	100.0	[33.0,100.0]	0.0	100.0	100.0	[100.0,100.0]	0.0	100.0	0.0256
Energy & Fatigue	47.0	[33.0,73.0]	13.3	100.0	57.0	[47.0,67.0]	20.0	80.0	0.1588
Wellbeing	65.0	[56.0,76.0]	16.0	96.0	68.0	[52.0,76.0]	36.0	84.0	0.6679
Social Function	75.0	[50.0,100.0]	25.0	100.0	88.0	[63.0,100.0]	25.0	100.0	0.2827
Bodily Pain	88.0	[68.0,100.0]	25.0	100.0	88.0	[74.0,100.0]	42.5	100.0	0.5754
General Health	56.0	[31.0,75.0]	15.0	87.5	63.0	[42.0,75.0]	6.25	81.3	0.3073

Abbreviations: IQR, interquartile range expressed as [1st quartile, 3rd quartile].

## Discussion

This is the first observational study evaluating patients’ adherence, satisfaction, and health related quality of life in WD patients before and after a change in treatment. The study addresses an important unmet need of WD patients, which is the adherence to treatment over a lifespan. Our data suggests that switching therapy during the maintenance phase to TETA 4HCl in adults enhances their self-reported adherence, mental and physical wellbeing with less variation in urinary copper excretion.

The data confirms the feasibility of switching patients from a range of therapies including penicillamine, TETA 2HCl or zinc salts, or a combination of chelator plus zinc, to TETA 4HCl. These data are aligned with observations from the CHELATE trial (Schilsky et al., 2022), which demonstrated non-inferiority of TETA 4HCl compared to DPA, real world studies of therapeutic switches to TETA 4HCl (Mohr et al., 2023; Woimant et al., 2022) which support the efficacy and tolerability of TETA 4HCl. Treatment adherence is widely recognized as an important factor determining a favourable WD prognosis (Roberts and Schilsky, 2023); conversely, non-adherence harms the clinical outcomes (Masełbas et al., 2019).

The predictive factors of poor adherence in WD have not been fully elucidated, and the reasons for poor adherence can change over time (Jacquelet et al., 2021). Identifying the most suitable therapy for each patient and balancing treatment efficacy with individual patient circumstances, including drug satisfaction and the impact on quality of life, through a multidisciplinary approach may be necessary to improve oral drug intake without interruptions.

To ensure methodological robustness, all essential methods to assess adherence, including pill count, validated self-report questionnaires, and clinical measurements of UCE values, were implemented, due to the limitations of each tool. The study population was highly educated and mostly employed or retired and showed a general improved adherence when assessed by all three methods used, although not always aligned for a given patient.

For pill count, 64% of the sample reported an adherence rate above 80%, while 76% (19 patients) reported an adherence rate above 60%. Interestingly, those diagnosed for over 20 years showed higher adherence to pill counting.

This is consistent with what was observed in the Jacquelet et al. cohort published in 2021, which examined 139 patients treated with DPA, TETA 2HCl and zinc salts (Jacquelet et al., 2021).

When switching from zinc, DPA or combination therapy, a shift to greater UCE values in the therapeutic range was observed. In particular, increased number of patients with UCE values in the therapeutic range was observed in the RP cohort from ST to T0, namely after the switch to TETA 4HCl but before the study started (namely, with no “study effect”). Additionally, a correlation between adherence measured by pill count and adherence measured by UCE values demonstrates the effectiveness of the new TETA 4HCl formulation.

Regarding self-reported adherence values (MMAS), an improvement was observed (from 14 adherent patients at T0 to 22 patients with self-declaring adherence at T1). However, no significant correlations were found with the other two adherence measurement methods.

Regardless of the measurement method, the improved adherence could be attributed in part to the effects of the close surveillance during the study and in part to the better tolerability and ease of use of the new TETA 4HCl formulation, as evidenced by the results of the PESaM questionnaire. These results suggest improvements in treatment satisfaction, with a statistically significant change observed in the “ease of use” domain of the PESaM questionnaire.

Furthermore, there was a strong reduction in the number of patients self-reporting adverse events after switching to TETA 4HCl, and no new adverse events were reported by patients at T1. Treatment with TETA 4HCl was well tolerated, with regression or improvement of adverse events induced by previous therapies and no patient reporting drug-related side effects.

The QoL results suggest improvements and, despite the high values at baseline, the two domains “Physical Functioning” and “Role Emotional” show statistically significant changes.

The benefits of TETA4HCl, in terms of adherence and emotional and physical health, were observed within 3 months of switching in the P cohort and were sustained for up to 12 months in the RP cohort. This could be attributed to the high-quality care provided at the three centres, which are referral centres in Italy for adult patients with WD.

However, technological advancements, such as a new drug formulation providing better bioavailability (Weigand and Corsini, 2022), could also have contributed to improving adherence and patient satisfaction. The posology (pill burden and frequency with potentially fewer restrictions around meals) and perceived convenience of blister pack, maintained at room temperature, combined with better tolerability of TETA 4HCl may have contributed to self-reporting on health-related quality of life, with the most significant benefits observed in domains related to the ability to manage daily activities, vitality, social functioning, and general health.

One limitation of the study is the small number of enrolled patients, consequently limiting the generalizability of the results. This limited sample size was partly due to the impact of the COVID-19 pandemic on study approval and execution. These factors, along with the small sample size and different MMAS tools, limit the generalizability of the findings.

Another limitation is the interval (3 months) between T0 and T1, which may not be predictive of continuing adherence over longer periods of time, an important factor in chronic diseases. The long-term benefits of TETA 4HCl on adherence requires further studies.

While we opted for the generic SF-36 to assess quality of life, given its broad applicability and validation across diseases, we acknowledge that WD-specific instruments including the Unified Wilson Disease Rating Scale (UWDRS) (Zhan et al., 2024) or EuroQoL-5D-5L (Mariño et al., 2023) have also been applied in recent studies. A 2021 systematic review on patient-reported outcome measures in WD (Balijepalli et al., 2021) further highlights the evolving landscape of tools in this area.

## Conclusion

Our study suggests potential benefits of TETA 4HCl in improving treatment adherence, patient satisfaction, and quality of life, though interpretations should remain cautious given methodological limitations.

Furthermore, long-term studies involving large sample sizes are warranted to confirm that the observed benefits extend over time. Overall, these findings add to the expanding body of evidence supporting the integration of patient-reported outcomes, such as treatment satisfaction and quality of life, into clinical research for rare diseases, aligning with the growing emphasis placed by regulatory authorities and health technology assessment bodies on patient-centered endpoints.

## Data Availability

The raw data supporting the conclusions of this article will be made available by the authors, without undue reservation.

## Abbreviations

ICMJE, International Committee of Medical Journal Editors; IQR, Interquartile Range; MMAS, Morisky Medication Adherence Scale; P, Prospective; PESaM, Patient Experiences and Satisfaction with Medication; RP, Retrospective-prospective; RSV, Respiratory syncytial virus; SD, Standard deviation; SF, Short Form; ST, Switch time; QoL, Quality of life; UCE, Urinary Copper Excretion; WD, Wilson disease.
